# Supplementary material for: Concentration of Selected Macronutrients and Toxic Elements in the Blood in Relation to Pain Severity and Hydrogen Magnetic Resonance Spectroscopy in People with Osteoarthritis of the Spine
Source: Int J Environ Res Public Health. 2022 Sep 9;19(18):11377. doi: 10.3390/ijerph191811377 (PMC9517490; doi:10.3390/ijerph191811377)
Supplement: Supplementary file 1 [file ijerph-19-11377-s001.zip › ijerph-1839159-supplementary.pdf]

nutrients MDPI

Supplementary Material

Concentration of Selected Macronutrients and Toxic Elements in the Blood in Relation to Pain Severity and Hydrogen Magnetic Resonance Spectroscopy in People with Osteoarthritis of the Spine

Marta Jakoniuk [1,*], Jan Kochanowicz [2], Agnieszka Lankau [3], Marianna Wilkiel [2] and Katarzyna Socha [4]

[1] Department of Invasive Neurology, Medical University of Białystok, M. Skłodowskiej-Curie 24a Street, 15-276 Białystok, Poland
[2] Department of Neurology, Medical University of Białystok, M. Skłodowskiej-Curie 24a Street, 15-276 Białystok, Poland
[3] Department of Integrated Medical Care, Medical University of Białystok, M. Skłodowskiej-Curie 7A Street, 15-096 Białystok, Poland
[4] Department of Bromatology, Medical University of Białystok, Mickiewicza 2D Street, 15-222 Białystok, Poland
* Correspondence: jakoniukmarta@gmail.com; Tel.: +48-85-748-21-22

Table S1. The concentration of calcium in the serum of patients with osteoarthritis of the spine and healthy people.

Ca (mg/L); RV: 86.2–102.2 mg/L				
**Group**	**Examined Group**		**Control Group**	
**Sex**	**F (a)**	**M (b)**	**F (c)**	**M (d)**
**Mean ± SD (min–max)**	91.20 ± 11.99 (72.97–119.40)	88.65 ± 10.15 (70.13–116.14)	92.42 ± 8.92 (76.11–110.45)	92.68 ± 121.92 (74.88–114.96)
**Median (Q_1–Q_3)**	90.50 (79.97–99.17)	91.26 (79.57–95.24)	92.28 (84.85–98.76)	90.80 (86.03–98.27)
**Smoking**	**Smokers (a)**	**Non-smokers (b)**	**Smokers (c)**	**Non-smokers (d)**
**Mean ± SD (min–max)**	86.99 ± 10.16 (70.13–105.33)	89.01 ± 11.36 (73.24–119.40)	93.85 ± 9.73 (81.41–107.71)	94.03 ± 10.23 (76.11–111.96)
**Median (Q_1–Q_3)**	85.80 (77.97–95.45)	90.71 (78.99–96.85)	93.0 (86.40–101.60)	95.44 (84.88–99.12)
**All persons (total)**				
**Mean ± SD (min–max)**	90.17 ± 11.28 (70.13–119.40)		92.50 ± 9.95 (74.88–114.96)	
**Median (Q_1–Q_3)**	90.92 (79.97–97.10)		91.73 (84.88–98.40)	

RV—reference value; F—female; M—male, SD—standard deviation; min—minimum; max—maximum; Q1—lower quartile; Q3—upper quartile; no significant differences in the Mann Whitney U-test.

Table S2. The concentration of lead in the blood of patients with osteoarthritis of the spine and healthy people.

Pb (μg/L); RV < 100 μg/L				
**Group**	**Examined Group**		**Control Group**	
**Sex**	**F (a)**	**M (b)**	**F (c)**	**M (d)**
**Mean ± SD**	40.23 ± 17.17	52.52 ± 69.13	35.55 ± 19.59	37.37 ± 13.25
**(min–max)**	(18.07–94.03)	(24.24–436.76)	(5.90–74.88)	(14.34–54.29)
**Median**	36.91	39.1	34.44	38.96
**(Q_1–Q_3)**	(28.25–46.76)	(30.25–50.75)	(17.66–51.06)	(26.68–50.54)
**Smoking**	**Smokers (a)**	**Non-smokers (b)**	**Smokers (c)**	**Non-smokers (d)**
**Mean ± SD**	54.55 ± 81.92	36.28 ± 11.57	29.49 ± 17.45	37.19 ± 17.34
**(min–max)**	(25.15–436.76)	(18.07–61.39)	(5.90–52.19)	(12.86–74.88)
**Median**	39.58	35.28	30.12	33.52
**(Q_1–Q_3)**	(29.37–43.99)	(27.58–43.45)	(10.13–44.80)	(28.82–46.47)
**All persons (total)**				
**Mean ± SD**	45.52 ± 47.18		36.10 ± 17.77	
**(min–max)**	(18.07–436.76)		(5.90–74.88)	
**Median**	37.74		35.36	
**(Q_1–Q_3)**	(28.72–47.85)		(20.07–50.54)	

RV—reference value; F—female; M—male, SD—standard deviation; min—minimum; max—maximum; Q1—lower quartile; Q3—upper quartile; no significant differences in the Mann Whitney U-test.

Table S3. The concentration of mercury in the blood of patients with osteoarthritis of the spine and healthy people.

Hg (μg/L); RV < 7.2 μg/L				
**Group**	**Examined Group**		**Control Group**	
**Sex**	**F (a)**	**M (b)**	**F (c)**	**M (d)**
**Mean ± SD**	1.10 ± 0.86	1.38 ± 1.03	1.32 ± 0.66	1.54 ± 0.84
**(min–max)**	(0.23–5.0)	(0.19–5.65)	(0.46–3.16)	(0.87–3.34)
**Median**	0.82	1.06	1.11	1.29
**(Q_1–Q_3)**	(0.23–5.0)	(0.86–1.61)	(0.87–1.61)	(0.97–1.74)
**Smoking**	**Smokers (a)**	**Non-smokers (b)**	**Smokers (c)**	**Non-smokers (d)**
**Mean ± SD**	1.28 ± 1.47	1.18 ± 0.99	1.10 ± 0.50	1.57 ± 0.80
**(min–max)**	(0.23–3.99)	(0.33–5.65)	(0.46–1.74)	(0.75–3.34)
**Median**	1.08	0.93	1.1	1.35
**(Q_1–Q_3)**	(0.72–1.6)	(0.44–1.59)	(0.75–1.46)	(0.94–2.06)
**All persons (total)**				
**Mean ± SD**	1.21 ± 0.94		1.37 ± 0.70	
**(min–max)**	(0.19–5.65)		(0.46–3.34)	
**Median**	1.01		1.25	
**(Q_1–Q_3)**	(0.58–1.60)		(0.88–1.61)	

RV—reference value; F—female; M—male, SD—standard deviation; min—minimum; max–maximum; Q1—lower quartile; Q3—upper quartile; no significant differences in the Mann Whitney U-test.

Table S4. Correlation coefficients between age, BMI, VAS and concentrations of mineral components in the serum (Ca, Mg), blood (Pb, Cd, Hg).

	Age	BMI	VAS
**Ca (mg/L)**	0.146	0.275	−0.102
**Mg (mg/L)**	0.005	−0.151	−0.058
**Pb (μg/L)**	0.33 **	0.42 **	−0.073
**Cd (μg/L)**	0.022	0.06	0.079
**Hg (μg/L);**	−0.054	0.112	0.110

BMI—Body Mass Index; VAS—Visual Analogue Scale; **—statistically significant correlation ($p < 0.01$) between age, BMI and blood Pb concentration of the study group in the Spearman rank test.

Table S5. Correlation coefficients between age, BMI, VAS and fat/water ratio in selected structures of the spine.

	Age	BMI	VAS
**Ratio L1**	0.066	0.043	0.167
**Ratio L5**	0.159	0.102	−0.105
**Ratio L4/5**	0.204	0.187	−0.197

BMI—Body Mass Index; VAS—Visual Analogue Scale; correlations not statistically significant.

Table S6. Correlation coefficients between concentrations of mineral components in the serum (Ca, Mg), blood (Pb, Cd, Hg) and fat/water ratio in selected structures of the spine.

	Ratio L1	Ratio L5	Ratio L4/5
**Ca (mg/L)**	−0.03	0.093	0.119
**Mg (mg/L)**	0.089	0.068	0.22
**Pb (μg/L)**	−0.181	−0.189	0.214 *
**Cd (μg/L)**	0.006	−0.114	0.214
**Hg (μg/L)**	0.166	−0.122	0.071

*—statistically significant correlation ($p < 0.05$) between blood Pb concentration and L4/L5 ratio of the study group in the Spearman rank test.
